# Synthetic Cannabinoid Use and Sports‐Related Concussion Risk Among US Adolescents: Implications for School Health Screening and Prevention

**DOI:** 10.1111/josh.70112

**Published:** 2026-01-05

**Authors:** Saurabh Kalra, Nandakumar Nagaraja, Deepak Kalra

**Affiliations:** ^1^ Miller School of Medicine University of Miami Miami Florida USA; ^2^ College of Medicine Pennsylvania State University Hershey Pennsylvania USA; ^3^ Cleveland Clinic Weston Florida USA

**Keywords:** adolescent health, school‐based screening, sports injury, substance use prevention, synthetic cannabinoids, traumatic brain injury

## Abstract

**Background:**

Synthetic cannabinoids (SCs), such as Spice and K2, appeal to adolescents because they are inexpensive and often evade standard drug tests. We assessed associations between SC use and sports‐related traumatic brain injuries (TBIs) among US adolescents.

**Methods:**

We analyzed nationally representative Youth Risk Behavior Survey data from 2017–2021 (*n* = 27,482). Sports‐related TBIs were defined as self‐reported concussion in the past year. SC use was defined as lifetime use. Weighted multivariable logistic regression assessed the associations, adjusting for demographic and behavioral factors.

**Results:**

Overall, 6.0% reported SC use, and 13% reported a sports‐related TBI. TBI prevalence was higher among SC users (22.9%) than non‐users (12.4%). SC use was associated with increased odds of a sports‐related TBI (AOR 1.48, 95% CI 1.30–1.70). Other significant predictors included boys vs. girls (AOR 1.38, 95% CI 1.28–1.49), Black race vs. White (AOR 1.26, 95% CI 1.13–1.40), current alcohol use (AOR 1.75, 95% CI 1.61–1.91), marijuana use (AOR 1.16, 95% CI 1.05–1.28), depressive symptoms (AOR 1.21, 95% CI 1.11–1.32), and suicidal ideation (AOR 1.14, 95% CI 1.03–1.25).

**Implications for School Health Policy, Practice, and Equity:**

Incorporating SC‐use screening into sports physicals and improving equitable access to concussion education may help reduce preventable injuries.

**Conclusions:**

SC use is associated with elevated sports‐related concussion risk among adolescents.

## 1 Introduction

Traumatic brain injury (TBI), including concussion, is a major cause of morbidity among US adolescents, particularly in the context of sports participation [1]. However, the role of modifiable risk factors—especially emerging drug use trends—has received less attention, despite the growing recreational use of synthetic cannabinoids (SCs) among youth athletes. SCs, often marketed as “Spice” or “K2,” appeal to adolescents due to their low cost, deceptive marketing, and undetectability on standard drug screens [2, 3]. These compounds, designed to mimic the psychoactive effects of delta‐9‐tetrahydrocannabinol (THC), are often portrayed as “safe” and “legal” alternatives to cannabis [4].

Globally, lifetime prevalence of SC use among adolescents ranges from 2% to 6%, with higher estimates reported in North America and Europe [2, 5]. In the United States, approximately 6.5% of high‐school students reported ever using SCs [6]. National surveillance data show that SC use among US adolescents peaked in the early 2010s, when national lifetime use reached about 10%–11% before declining to current levels near 6% [6, 7, 8]. Despite this decline, the ongoing emergence of new SC analogs and online marketing continues to sustain availability and use among youth [9].

Although SCs may not cause concussions directly, they can dangerously impair cognitive and motor functions critical for sports. By reducing coordination, judgment, and reaction time, SCs may elevate the risk of sustaining a TBI during athletic activities [10, 11]. Adverse effects include confusion, paranoia, hallucinations, muscle spasms, seizures, and in rare cases, life‐threatening conditions such as cardiac arrhythmias—many of which could increase injury susceptibility [12, 13]. Moreover, because SCs often evade routine drug tests and may dull pain or dizziness, athletes may unknowingly remain in play when medical attention is warranted [14]. These risks are heightened in youth with prior concussions or in contact sports [15].

Despite increasing concern, research examining the relationship between SC use and sports‐related concussions is limited [16]. Prior studies have highlighted associations between concussion and factors like alcohol and marijuana use, depression, overexertion, sleep disruptions, and sports type [17, 18, 19]. This study investigates the association between SC use and sport‐related TBIs in US adolescents. Given their role in return‐to‐play decisions and injury prevention, these findings have important implications for school health providers [20, 21, 22]. We hypothesize that adolescent athletes who use SCs may be at a higher risk of sustaining TBIs compared to their non‐using peers.

## 2 Methods

### 2.1 Sampling

We utilized data from the National High School Youth Risk Behavior Surveys (YRBS) conducted in 2017, 2019, and 2021. The YRBS is a biennial survey by the Centers for Disease Control and Prevention (CDC) that monitors health risk behaviors among adolescents in the United States [8]. It employs a 3‐stage cluster sampling technique to gather nationally representative data from both public and private schools across all 50 states and the District of Columbia. For this study, publicly available data from the 2017 (*n* = 14,765), 2019 (*n* = 13,677), and 2021 (*n* = 17,232) YRBS administrations were used. Response rates for these surveys ranged from 57.5% to 61.1%.

Out of the initial 45,674 respondents, 18,192 were excluded due to missing data on key outcomes (sports‐related concussions, *n* = 4308), exposures (synthetic marijuana, *n* = 7069), and demographic variables (sex, *n* = 344; grade, *n* = 122; race, *n* = 539), and other covariates such as feelings of sadness or hopelessness (*n* = 414), suicidal ideation (*n* = 101), sleep (*n* = 2564), alcohol use (*n* = 2519), and marijuana use (*n* = 212). The final analytic sample comprised 27,482 adolescents. The final sample was comparable to the full cohort, suggesting minimal bias.

### 2.2 Study Measures

**Outcome variable**: Concussion history was assessed through the question: “During the past 12 months, how many times have you had a concussion from playing sports or being physically active?” Responses were recorded across several options, including “0 times,” “1 time,” “2 times,” and “3 or more times.” Prior to this question, the YRBS defined “concussion” as a mild traumatic brain injury (TBI) caused by a blow to the head or body, resulting in temporary disruption of brain function. Symptoms may include headache, confusion, dizziness, or loss of consciousness. Based on these responses, YRBS created a dichotomous variable with response options: “Yes” (for students who reported having a concussion in the past 12 months) and “No” (for those reporting none).

**Exposure (SC use)**: Synthetic marijuana use was assessed by asking, “During your life, have you ever used synthetic marijuana (also called ‘K2’, ‘Spice’, ‘fake weed’, ‘King Kong’, ‘Yucatan Fire’, ‘Skunk’, or ‘Moon Rocks’, one or more times)?” Responses were recorded as either “Yes” or “No.”

**Covariates**: Demographic variables, including sex assigned at birth, grade, and race/ethnicity, were also controlled for. In addition, indicators such as alcohol use in the past month, marijuana use in the past month, average sleep of less than 8 h per night, feelings of sadness or hopelessness in the past year, and survey year were included as control variables. These factors were selected based on their theoretical and empirical relevance to both the independent and dependent variables [17, 18].

### 2.3 Statistical Methods

Descriptive statistics and logistic regression were used to evaluate associations between sports‐related TBI and SC use. Differences in descriptive statistics were assessed using the adjusted F statistic, a modified version of the Rao‐Scott chi‐square test. Logistic regression models were employed to assess the associations and were adjusted for demographic and health behavior factors, as previously mentioned. A backward elimination method (p‐value > 0.20 for removal) was applied to refine the models and retain only significant predictors. A sensitivity analysis was conducted using unadjusted logistic regression, which included observations initially excluded due to missing covariate data (*n* = 32,943). Multicollinearity was evaluated by calculating variance inflation factors (VIF), with all VIFs below 1.54, indicating no significant multicollinearity. All analyses accounted for the YRBS complex survey design using sampling weights, strata, and primary sampling unit variables with Taylor‐series linearization. Because school‐level identifiers are not available in the public‐use data, multilevel modeling was not possible; the design‐based approach provides unbiased, nationally representative estimates.

Missing data were handled using a listwise deletion approach, excluding respondents with incomplete information on key variables. Overall, missingness was < 10% for most variables and was not associated with the outcome. Multiple imputation was not used because the YRBS is a large, cross‐sectional survey with minimal missingness; listwise deletion is appropriate under these conditions for complex‐survey analyses [23]. All analyses were performed using STATA 18.0 software (StataCorp). We used publicly available, de‐identified data; the study was deemed exempt from IRB review.

## 3 Results

Among 27,482 adolescents, 13% reported experiencing a sports‐related TBIs in the past year. Additionally, 6.0% reported SC use, while past‐month alcohol and marijuana use were reported by 27.5% and 18.2%, respectively. Additionally, 36.7% reported feelings of sadness or hopelessness, 19.2% considered suicide, and 76.7% reported sleeping less than 8 h per night (Table 1). SC use was more prevalent among those with sports‐related TBIs compared to those without (10.8% vs. 5.3%). Boys more commonly reported a history of sports‐related TBIs than girls (55.2% vs. 44.8%). Adolescents with sports‐related TBIs also more commonly reported past‐month alcohol use (40.0% vs. 25.5%), marijuana use (26.2% vs. 17.0%), feelings of sadness or hopelessness (41.5% vs. 35.8%), and suicidal ideation (23.7% vs. 18.6%) (Table 1).

**TABLE 1:** Characteristics of study population, high‐school students, Youth Risk Behavior Survey (YRBS) (2017–2021) (*n* = 27,482), United States (Weighted Prevalence, % (se)).

Independent variables	All adolescents	Adolescents with no history of concussion from playing sports, past year (*n* = 23,995)	Adolescents with a history of concussion from playing sports, past year (*n* = 3588)	*p* ^c^
Sex: Girls	50.0 (0.6)	50.8 (0.7)	44.8 (1.2)	< 0.001
Boys	50.0 (0.6)	49.2 (0.7)	55.2 (1.2)	
Grade: 9th grade	25.9 (0.5)	25.6 (0.5)	29.3 (1.1)	< 0.001
10th grade	25.1 (0.4)	25.2 (0.4)	25.1 (0.9)	
11th grade	24.8 (0.4)	24.5 (0.4)	26.0 (1.0)	
12th grade	24.2 (0.4)	24.6 (0.4)	19.7 (0.9)	
Race/Ethnicity: Non‐Hispanic White	53.5 (1.9)	53.1 (1.8)	53.8 (2.1)	0.417
Non‐Hispanic Black	10.9 (0.8)	11.2 (0.7)	12.7 (1.1)	
Hispanic/Latinos	24.4 (1.5)	24.4 (1.6)	23.7 (1.7)	
Non‐Hispanic Others	11.2 (0.9)	11.4 (0.9)	9.8 (0.8)	
Alcohol use, past month	27.5 (0.8)	25.5 (0.7)	40.0 (1.3)	< 0.001
Felt sad or hopeless, past year^b^	36.7 (0.7)	35.8 (0.8)	41.5 (1.2)	< 0.001
Marijuana use, past month	18.2 (0.6)	17.0 (0.6)	26.2 (1.2)	< 0.001
Sleep less than 8 h	76.7 (0.5)	76.6 (0.5)	77.0 (0.8)	0.738
Year: 2017	33.1 (4.7)	33.5 (4.8)	36.6 (5.1)	< 0.001
2019	31.7 (4.5)	30.8 (4.4)	34.1 (4.6)	
2021	35.2 (5.4)	35.6 (5.4)	29.4 (4.9)	
Synthetic marijuana use, ever^a^	6.0 (0.2)	5.3 (0.2)	10.8 (0.6)	< 0.001

*Note:* All analysis accounts for YRBS survey weights to be nationally representative for all adolescents.

^a^ Respondents were asked if they had ever used synthetic cannabinoids, also known as Spice, fake weed, K2, or Black Mamba.

^b^ Respondents were asked if, within the past year, they had ever felt so sad or hopeless almost every day for 2 weeks or more in a row that they stopped doing some usual activities.

^c^ *p*‐values are from the chi‐square test for independence, assessing associations with concussion history.

Multivariable logistic regression analyses showed that adolescents who reported SC use were more likely to have a history of sports‐related TBIs compared to non‐users (AOR 1.48, 95% CI 1.30–1.70) (Table 2). Other significant factors included being male (AOR 1.38, 95% CI 1.28–1.49), being Non‐Hispanic Black compared to Non‐Hispanic White (AOR 1.26, 95% CI 1.13–1.40), current alcohol use (AOR 1.75, 95% CI 1.61–1.91), marijuana use (AOR 1.16, 95% CI 1.05–1.28), feelings of sadness or hopelessness (AOR 1.21, 95% CI 1.11–1.32), and suicidal ideation (AOR 1.14, 95% CI 1.03–1.25). In a sensitivity analysis excluding variables with high missingness (sleep, suicidal ideation, and alcohol use), the association between SC use and sports‐related TBIs remained significant (AOR 1.80, 95% CI 1.61–2.02). The final model demonstrated adequate calibration (Hosmer–Lemeshow χ^2^(8) = 11.30, *p* = 0.19) and modest discrimination (area under the curve [AUC] = 0.62), explaining approximately 4% of the variance in sports‐related TBI risk (Nagelkerke pseudo‐*R* ^2^ = 0.038).

**TABLE 2:** Multivariable logistic regression analysis of sports‐related concussions and synthetic cannabinoid use among high school students, Youth Risk Behavior Survey (YRBS) 2017–2021, United States.

Independent variables	Sports‐related concussions (*n* = 27,482), AOR (95% CI)	*p*
Synthetic cannabinoids use^a^	1.48 (1.30–1.70)	< 0.001**
Sex: Girls	1.00 (Ref.)	—
Boys	1.38 (1.28–1.49)	< 0.001**
Grade: 9th grade	1.00 (Ref.)	
10th grade	0.86 (0.78–0.95)	< 0.001**
11th grade	0.79 (0.72–0.88)	< 0.001**
12th grade	0.64 (0.57–0.70)	< 0.001**
Race/ethnicity: Non‐Hispanic White	1.00 (Ref.)	—
Non‐Hispanic Black	1.26 (1.13–1.40)	0.002**
Hispanic/Latinos	0.97 (0.88–1.06)	0.431
Non‐Hispanic others	1.01 (0.89–1.13)	0.295
Alcohol use, past month	1.75 (1.61–1.91)	< 0.001**
Felt sad or hopeless, past year^b^	1.21 (1.11–1.32)	< 0.001**
Considered suicide, past year	1.14 (1.03–1.25)	< 0.001**
Marijuana use, past month	1.16 (1.05–1.28)	< 0.011*

*Note:* In addition to above covariates, analysis accounts for survey‐year and YRBS survey weights.

Abbreviations: AOR, adjusted odds ratio; CI, confidence interval.

^a^ Respondents were asked if they had ever used synthetic cannabinoids, also known as Spice, fake weed, K2, or Black Mamba.

^b^ Respondents were asked if, within the past year, they had ever felt so sad or hopeless almost every day for 2 weeks or more in a row that they stopped doing some usual activities.

* *p* < 0.05.

** *p* < 0.01.

## 4 Discussion

This nationally representative study is among the first to demonstrate an independent association between SC use and sports‐related concussions in adolescents, offering actionable insights for health providers. The association persisted after accounting for alcohol and marijuana use, depressive symptoms, sleep, and demographic factors, suggesting that SC use may reflect a distinct behavioral or neurocognitive vulnerability relevant to injury risk. These findings align with prior studies showing that substance use, emotional distress, and male sex are important correlates of concussion among high‐school adolescents [19]. We also observed higher odds of sports‐related concussion among non‐Hispanic Black adolescents compared with non‐Hispanic White adolescents, underscoring the importance of examining equity in both concussion risk and recognition within school settings.

SCs are often misperceived as harmless or legal alternatives to marijuana [2, 3, 24]. However, their unpredictable potency and neuropsychiatric effects—such as delayed reflexes, impaired judgment, and diminished awareness—can increase the risk of both initial and repeat TBIs, particularly in athletic settings [4]. These impairments may cause athletes to overlook symptoms or continue playing when they should be removed from activity, potentially leading to repeat TBIs.

TBIs in adolescents can lead to lasting cognitive, emotional, and behavioral consequences, including memory problems, mood changes, and heightened vulnerability to substance use [25]. These outcomes may adversely affect academic performance, social functioning, and long‐term health. Because adolescent brain development and risk‐taking behaviors intersect during this period, understanding modifiable contributors such as substance use is critical for injury prevention efforts.

Our findings contribute to a growing literature suggesting that emerging drugs, including SCs with rapidly shifting formulations, may represent an underrecognized risk in youth sports [12]. Continued surveillance and research are needed to assess how evolving SC products influence adolescent neurobehavioral health, to explore causal pathways, and to determine whether specific subgroups of youth athletes may be disproportionately affected.

### 4.1 Implications for School Health Policy, Practice, and Equity

Schools can reduce preventable injuries by incorporating brief SC‐use screening into existing health touchpoints, including pre‐participation physicals and post‐concussion evaluations [26]. Because SCs are often misperceived as safer alternatives to marijuana and can impair coordination and judgment, school nurses and athletic trainers may play a key role in identifying students at elevated neurological risk. Routine, confidential screening questions embedded in sports assessments could help uncover SC use that might otherwise go unreported, supporting early intervention and safer participation in school athletics [2, 3, 24, 27, 28].

These findings also highlight the need to strengthen equitable access to both substance‐use education and concussion assessment. Prior work shows that schools vary widely in concussion‐related policies, return‐to‐learn procedures, and availability of trained health personnel, contributing to disparities in detection and management [20, 21, 22]. Enhancing staff training on emerging substances like SCs, ensuring consistent application of concussion protocols across school populations, and expanding culturally responsive education on substance‐related risks may reduce preventable injuries and support healthier learning environments for all adolescents [20, 21, 22, 24].

### 4.2 Limitations

The cross‐sectional design of our study limits causal inference regarding the relationship between SC use and sports‐related TBI. Reliance on self‐reported data introduces potential recall and social desirability biases, as students may underreport SC use or concussions. Additionally, the dataset lacked detail on the frequency of SC use, concussion severity, and important confounders such as socioeconomic status and comorbid mental health conditions. The inclusion of YRBS cycles spanning pre‐ and post‐COVID‐19 years may also introduce temporal variability in behavioral patterns despite statistical adjustment for survey year. Future longitudinal research using objective measures (e.g., drug testing, clinical assessments) is needed to confirm these findings and explore causal pathways.

## 5 Conclusions

Adolescents who use SC are at higher risk of reporting a sports‐related concussion within the past year. These findings highlight the need for school health providers to recognize and screen for SC use when evaluating concussion risk and conducting prevention counseling. Early identification and targeted interventions may help reduce the future burden of neurological injury.

## Funding

This research received no external funding.

## Conflicts of Interest

The authors declare no conflicts of interest.

## Data Availability

The deidentified data supporting the findings of this study are publicly available from the Youth Risk Behavior Survey (YRBS), conducted by the Centers for Disease Control and Prevention, at https://www.cdc.gov/yrbs/index.html.

## References

[1] C. A. Taylor , “Traumatic Brain Injury‐Related Emergency Department Visits, Hospitalizations, and Deaths—United States, 2007 and 2013,” MMWR Surveillance Summaries 66 (2017): 1–16. 10.15585/mmwr.ss6609a1 PMC5829835 28301451

[2] R. Baweja , S. Mills‐Huffnagle , A. Jernigan , N. Chongtham , D. Waschbusch , and J. G. Waxmonsky , “Synthetic Marijuana: Assessment of Usage, Motivation and Associated Risks in Adolescent Substance Users,” Substance Use: Research and Treatment 18 (2024): 29768357241254258. 38764525 10.1177/29768357241254258 PMC11102655

[3] X. Liu , A. Villamagna , and J. W. Yoo , “The Importance of Recognizing Cannabinoid Hyperemesis Syndrome From Synthetic Marijuana Use,” Journal of Medical Toxicology 13, no. 2 (2017): 199–200, 10.1007/s13181-017-0612-x. 28353201 PMC5440325

[4] K. J. Debnam , S. Saha , and C. P. Bradshaw , “Synthetic and Other Drug Use Among High School Students: The Role of Perceived Prevalence, Access, and Harms,” Substance Use & Misuse 53, no. 12 (2018): 2069–2076. 29624111 10.1080/10826084.2018.1455699 PMC6136142

[5] Drugs UNOo , World Drug Report 2024 (Set of 3 Booklets) (Stylus Publishing, 2024).

[6] B. E. Hoots , “Alcohol and Other Substance Use Before and During the COVID‐19 Pandemic Among High School Students—Youth Risk Behavior Survey, United States, 2021,” MMWR Supplements 72, no. Suppl 1 (2023): 84–92. 37104552 10.15585/mmwr.su7201a10 PMC10156154

[7] K. Clements‐Nolle , T. Lensch , S. Larson , and W. Yang , “Prevalence and Correlates of Any and Frequent Synthetic Cannabinoid Use in a Representative Sample of High School Students,” Substance Use & Misuse 51, no. 9 (2016): 1139–1146. 27191966 10.3109/10826084.2016.1160121

[8] Centers for Disease Control and Prevention , Youth Risk Behavior Survey Data Summary & Trends Report: 2013–2023 (US Department of Health and Human Services, 2024).

[9] L. D. Johnston , R. A. Miech , M. E. Patrick , P. M. O'Malley , J. E. Schulenberg , and J. G. Bachman , Monitoring the Future National Survey Results on Drug Use, 1975–2022: Overview, Key Findings on Adolescent Drug Use (Institute for Social Research, 2023).

[10] A. Weinstein , P. Rosca , L. Fattore , and E. D. London , “Synthetic Cathinone and Cannabinoid Designer Drugs Pose a Major Risk for Public Health,” Frontiers in Psychiatry 8 (2017): 156, 10.3389/fpsyt.2017.00156. 28878698 PMC5572353

[11] K. Sarmiento , Z. Donnell , and R. Hoffman , “A Scoping Review to Address the Culture of Concussion in Youth and High School Sports,” Journal of School Health 87, no. 10 (2017): 790–804. 28876477 10.1111/josh.12552 PMC6211168

[12] J. Phillips , F. Lim , and R. Hsu , “The Emerging Threat of Synthetic Cannabinoids,” Nursing Management 48, no. 3 (2017): 22–30. 10.1097/01.NUMA.0000512504.16830.b6 28234762

[13] S. Chiappini , A. Mosca , A. Miuli , et al., “New Psychoactive Substances and Suicidality: A Systematic Review of the Current Literature,” Medicina 57, no. 6 (2021): 580, 10.3390/medicina57060580. 34204131 PMC8226910

[14] A. Maglaviceanu , M. Peer , J. Rockel , et al., “The State of Synthetic Cannabinoid Medications for the Treatment of Pain,” CNS Drugs 38, no. 8 (2024): 597–612. 38951463 10.1007/s40263-024-01098-9

[15] P. McCrory , W. Meeuwisse , J. Dvorak , et al., “Consensus Statement on Concussion in Sport—The 5th International Conference on Concussion in Sport Held in Berlin, October 2016,” British Journal of Sports Medicine 51, no. 11 (2017): 838–847. 28446457 10.1136/bjsports-2017-097699

[16] K. D. Mallory , L. Saly , A. Hickling , H. Colquhoun , E. Kroshus , and N. Reed , “Concussion Education in the School Setting: A Scoping Review,” Journal of School Health 92, no. 6 (2022): 605–618. 35259774 10.1111/josh.13156 PMC9311225

[17] L. DePadilla , G. F. Miller , S. E. Jones , and M. J. Breiding , “Substance Use and Sports‐ or Physical Activity‐Related Concussions Among High School Students,” Journal of School Nursing 38, no. 6 (2022): 511–518, 10.1177/1059840520977319. PMC9514098 33267719

[18] K. Sarmiento , G. F. Miller , and S. E. Jones , “Sports‐ or Physical Activity‐Related Concussions and Feelings of Sadness or Hopelessness Among U.S. High School Students: Results From the 2017 Youth Behavior Risk Survey,” Journal of School Nursing 38, no. 2 (Apr 2022): 203–209, 10.1177/1059840520945389. PMC7881043 32787613

[19] D. S. Younger , “Mild Traumatic Brain Injury and Sports‐Related Concussion,” Handbook of Clinical Neurology 196 (2023): 475–494. 37620086 10.1016/B978-0-323-98817-9.00001-6

[20] V. H. Lyons , M. Moore , R. Guiney , et al., “Strategies to Address Unmet Needs and Facilitate Return to Learn Guideline Adoption Following Concussion,” Journal of School Health 87, no. 6 (2017): 416–426. 28463445 10.1111/josh.12510 PMC8570132

[21] K. McAvoy , B. Eagan‐Johnson , R. Dymacek , S. Hooper , M. McCart , and J. Tyler , “Establishing Consensus for Essential Elements in Returning to Learn Following a Concussion,” Journal of School Health 90, no. 11 (2020): 849–858. 32939780 10.1111/josh.12949

[22] L. DePadilla, PhD , G. F. Miller, PhD , M. Everett Jones, PhD , and S. Jd , “Characteristics of Schools With Youth Sports Concussion‐Related Educational Policies and Practices,” Journal of School Health 90, no. 7 (2020): 520–526. 32350884 10.1111/josh.12900 PMC11600411

[23] S. G. Heeringa , B. T. West , S. G. Heeringa , and P. A. Berglund , Applied Survey Data Analysis (Chapman and Hall/CRC, 2017).

[24] A. B. M. Paul , L. Simms , S. Amini , and A. E. Paul , “Teens and Spice: A Review of Adolescent Fatalities Associated With Synthetic Cannabinoid Use,” Journal of Forensic Sciences 63, no. 4 (2018): 1321–1324. 29194599 10.1111/1556-4029.13704

[25] H. A. Shepherd , K. O. Yeates , N. Reed , et al., “Academic Accommodations for Middle and High School Students Following a Concussion: Perspectives of Teachers and School Administrators,” Journal of School Health 93, no. 12 (2023): 1099–1110. 37386759 10.1111/josh.13360

[26] J. J. Leddy , “Sport‐Related Concussion,” New England Journal of Medicine 392, no. 5 (2025): 483–493. 39879594 10.1056/NEJMcp2400691

[27] V. Filleul , F. d'Arripe‐Longueville , M. Garcia , et al., “Anti‐Doping Education Interventions in Athletic Populations: A Systematic Review of Their Characteristics, Outcomes and Practical Implications,” International Review of Sport and Exercise Psychology 18 (2024): 880–942.

[28] L. Goldberg and D. L. Elliot , “Preventing Substance Use Among High School Athletes: The ATLAS and ATHENA Programs,” in School Sport Psychology (Routledge, 2013), 63–87.

